# Streptococcal Infection as a Major Historical Cause of Stuttering: Data, Mechanisms, and Current Importance

**DOI:** 10.3389/fnhum.2020.569519

**Published:** 2020-11-09

**Authors:** Per A. Alm

**Affiliations:** Department of Neuroscience, Uppsala University, Uppsala, Sweden

**Keywords:** stuttering, infection, Group A beta-hemolytic streptococci, *Streptococcus pyogenes*, autoimmunity, inflammation, epidemiology, basal ganglia

## Abstract

Stuttering is one of the most well-known speech disorders, but the underlying neurological mechanisms are debated. In addition to genetic factors, there are also major non-genetic contributions. It is here proposed that infection with group A beta-hemolytic streptococcus (GAS) was a major underlying cause of stuttering until the mid-1900s when penicillin was introduced in 1943. The main mechanism proposed is an autoimmune reaction from tonsillitis, targeting specific molecules, for example within the basal ganglia. It is here also proposed that GAS infections may have continued to cause stuttering to some extent, to the present date, though more rarely. If so, early diagnosis of such cases would be of importance. Childhood cases with sudden onset of stuttering after throat infection may be particularly important to assess for possible GAS infection. The support for this hypothesis primarily comes from three lines of argument. First, medical record data from the 1930s strongly indicates that there was one type of medical event in particular that preceded the onset of childhood stuttering with unexpected frequency: diseases related to GAS throat infections. In particular, this included tonsillitis and scarlet fever, but also rheumatic fever. Rheumatic fever is a childhood autoimmune sequela of GAS infection, which was a relatively widespread medical problem until the early 1960s. Second, available reports of changes of the childhood prevalence of stuttering indicate striking parallels between stuttering and the incidence of rheumatic fever, with: (1) decline from the early 1900s; (2) marked decline from the introduction of penicillin in the mid 1940s; and (3) reaching a more stable level in the 1960s. The correlations between the data for stuttering and rheumatic fever after the introduction of penicillin are very high, at about 0.95. Third, there are established biological mechanisms linking GAS tonsillitis to immunological effects on the brain. Also, a small number of more recent case reports have provided further support for the hypothesis linking stuttering to GAS infection. Overall, it is proposed that the available data provides strong evidence for the hypothesis that GAS infection was a major cause of stuttering until the mid-1900s, interacting with genetic predisposition.

## Introduction

### Background

Stuttering is one of the most common speech disorders, but the specific underlying neurological mechanisms are elusive. Several different mechanisms and pathologies can likely result in similar symptoms. The core symptoms manifest as intermittent loss of volitional control of the speech movements, resulting in repetitions, prolongations, or blocks.

The available data on the prevalence of stuttering suggests that there was a substantial reduction in incidence from the early 1900s to the 1960s. Thereafter, the incidence level appears to have become more stable (see review and discussion below). So far there is no established well-founded explanation of this likely historical decline of stuttering. Extensive medical record data from children before and after the onset of stuttering is available from the 1930s, thanks to the work of Dr. Mildred Berry (Berry, 1938). This is probably the largest and most thorough investigation of medical conditions related to childhood stuttering published to date. It was based on Chicago hospital medical records and included 430 children who stuttered and 462 children without stuttering. An inclusion criterion was that only medical records that appeared to be complete until age 9 were included. What stands out in the result is the high frequency of infections before the onset of stuttering, in particular infections related to *Streptococcus pyogenes*, such as tonsillitis, scarlet fever, and cervical abscess. *Streptococcus pyogenes* is the dominant strain of *Group A beta-hemolytic streptococci* (GAS). GAS infections are known or suspected to be involved in the causal background of a range of autoimmune symptoms, including neurological symptoms related to the basal ganglia, such as Sydenham’s chorea. GAS infections have later been linked to *pediatric autoimmune neuropsychiatric disorders associated with streptococcal infections* (PANDAS; Swedo et al., 1998).

In particular, GAS is linked to *acute rheumatic fever*, a serious systemic autoimmune disorder, which may affect a large number of body tissues. In this context, it is of interest that the incidence of rheumatic fever has declined dramatically in the industrial world, from the late 1800s to early 1960, from being a major medical problem to a rare condition. The decline can partly be explained by the introduction of effective antibiotics in the 1940s, but the decline began earlier. Rheumatic fever is still to a large extent a poorly understood condition, both regarding its pathophysiology and the causes of its decline. In summary, both stuttering and rheumatic fever appear to have shown a marked decline in incidence during the same period, from about the early 1900s to early 1960.

### Purpose and Article Overview

The purpose of this article is to analyze and discuss the possibility that the development of stuttering in some children is related to GAS infection, and that this has been a major causal factor earlier in history. It is preliminarily postulated by the author that such an effect could be the result of an autoimmune reaction to some specific molecular structures of the brain, in parallel to Sydenham’s chorea. Further, another preliminary assumption is that the affected brain structures involve the speech motor circuits of the basal ganglia. Because the impact of GAS infections was stronger before effective antibiotics were available, it is important to analyze data from that time and to analyze the period after antibiotics were introduced. If GAS infection has been a major factor it can be predicted that the introduction of penicillin would result in a decline of the prevalence of stuttering. Therefore, a large portion of the article is focused on the analysis of historical data, but also more recent case reports are reviewed.

Relevant literature and data will be reviewed and analyzed, regarding:

1) *the properties of the GAS bacteria*, including the known diseases and autoimmune reactions following GAS infections, in particular focusing neurological symptoms.
2) *modern case reports* of stuttering suspected to be related to GAS infections.
3) reports from the 1930s regarding *the medical history of children who stuttered*.
4) the historical *incidence of stuttering in relation to the incidence of rheumatic fever*
5) the historical *incidence of stuttering in relation to the introduction of penicillin*.

The evaluation of the combined weight of these different aspects may be viewed in terms of “triangulation,” as used in mixed methods research (Howe, 2012). This concept refers to the combination of different types of data in the study of a specific phenomenon and is borrowed from the practice in the navigation to determining the location of a specific point based on observations from at least three angles. Transferred to research, the concept implies that multiple types of data, pointing towards the same interpretation, becomes more reliable than each type of data in isolation.

## Theories on the Neurobiological Substrate of Stuttering

The neurobiological underpinnings of stuttering is a topic of ongoing research, and a multitude of theories have been proposed. It is known that there is a relatively strong genetic influence (Kraft and Yairi, 2012; Frigerio-Domingues and Drayna, 2017), but there is also evidence for non-genetic causal factors (Drayna et al., 1999; Alm and Risberg, 2007). Regarding the structural basis of stuttering, at least three possible mechanisms are of current interest: (1) theories of abnormal cerebral lateralization; (2) theories of white matter disconnection; and (3) theories of basal ganglia disorder.

### Cerebral Lateralization

Theories of cerebral lateralization, and hemispheric conflict, have a long history since first proposed by Stier (1911). This line of thinking was revived in the report by Jones (1966), of stuttering disappearing after cerebral lesions. Since then a wide range of studies, with different methodologies, have reported reduced left-hemisphere dominance for speech-related functions in persons who stutter. A modern version of the cerebral-hemisphere-conflict-model was presented by Neef et al. (2016). Indications of primary deficits of left hemisphere cortical speech-related regions in children who stutter have been reported by, for example, Garnett et al. (2018).

### White Matter Disconnection

The theory of white matter disconnection as a key aspect of stuttering was first proposed by Sommer et al. (2002), based on their findings from brain imaging. Their results indicated a localized impairment of the white matter structure, below the left hemisphere sensorimotor representations of the larynx and the tongue. Similar left hemisphere white matter impairments have later been reported by several groups, as summarized in Watkins (2011). Recently Han et al. (2019) proposed a genetic mechanism for white matter impairment in stuttering.

### The Basal Ganglia Loops

Historical theories by the German physicians Sahli and Schilder in the 1920s linked stuttering to the functions of the basal ganglia (Freund, 1966). The possible relationship between stuttering and the basal ganglia is discussed in Alm (2004) and later updated by Chang and Guenther (2020). Empirical findings are reported in, for example, Wu et al. (1995, 1997)^1^, Giraud et al. (2008), Lu et al. (2010), Tani and Sakai (2011), and Theys et al. (2013). The core proposal is that stuttering is related to a malfunction of the basal ganglia loop for the initiation of speech motor programs. One important line of argument for the involvement of the basal ganglia emerges from studies of stuttering with onset after known brain lesions. In a study of people who acquired stuttering after injury in wartime, the only gray matter structures that were statistically linked to stuttering were basal ganglia nuclei (Ludlow et al., 1987). A further indication of a link between stuttering and the basal ganglia can be seen from the effects of dopaminergic drugs: The functions of the basal ganglia are highly dependent on narrowly regulated dopamine activity, and the class of drugs that has shown the strongest modulating effect on stuttering, making it better or worse, are the dopaminergic drugs (Alm, 2004; Maguire et al., 2004). Stuttering also shows similarities with basal ganglia motor disorders, for example a combination of hypo- and hyperkinetic dysfunction, and temporary improvement of symptoms when providing external timing cues, such as a metronome.

## Properties of Disorders Linked to GAS Infection

In this section, the characteristics of the GAS bacteria will be reviewed, including various disorders that can follow from GAS infections. The focus will be on aspects of autoimmunity and in particular on neurological symptoms.

### The Group A Beta-Hemolytic Streptococcus (GAS)

GAS infections typically imply infection by the species *Streptococcus pyogenes* (pyogenes, from Greek: to produce pus^2^). “Beta-hemolytic” means that the bacteria produce *streptolysin*, a toxin that can result in a complete breakdown of red blood cells (hemolysis). It is important to emphasize that there are many other types of streptococci, which should not be confused with GAS. GAS infections can result in an exceptionally wide range of different symptoms, from mild to severe, including fatal. Scarlet fever and rheumatic fever are diseases specific to GAS infection, while other types of symptoms associated with GAS may also be caused by other types of bacteria, for example, pharyngitis, tonsillitis, and infections of the skin and other soft tissues (Ferretti and Köhler, 2017). At a global level, it is estimated that severe GAS infections lead to extensive disability and more than 500,000 deaths each year (Carapetis et al., 2005). There is currently no vaccine available, but development is in progress.

Many of the symptoms caused by GAS are effects of the large number of extracellular molecules that are produced by the bacteria (Ferretti and Köhler, 2017), and of autoimmune reactions. The production of extracellular molecules varies depending on the GAS strain and the host environment. An important characteristic of *Streptococcus pyogenes* is the production of “superantigens;” a family of peptides or small proteins with the ability to trigger massive unspecific activation of the immune system, with excessive release of T cell mediators and pro-inflammatory cytokines (Proft and Fraser, 2016).

Strains of GAS bacteria differ in their ability to avoid the host immune system and antibiotics. These mechanisms are not fully understood but have been proposed to include encapsulation by hyaluronic acid and the production of protective *biofilm* (Vyas et al., 2019), as well as the capacity to hide within host cells (Fiedler et al., 2015; Rohde and Cleary, 2016). It seems that the bacteria tend to shift between free-floating and more protected states (Vyas et al., 2019), making the duration of antibiotic treatment an important factor. It has been proposed that chronic tonsillar GAS infections may be temporarily asymptomatic as a result of biofilm protection or intracellular state (Allen and Miller, 2016; Rohde and Cleary, 2016). Macrolide antibiotics such as azithromycin have a higher ability to penetrate host cell walls than penicillin but have also a higher risk for the development of GAS resistance (Fiedler et al., 2015; Rohde and Cleary, 2016; Vyas et al., 2019).

The nature and virulence of the GAS bacteria that circulate in society appear to change quite dramatically over time, resulting in different panoramas of diseases and symptoms. For example, toward the end of the 1800s scarlet fever showed a mortality rate of 25–30%, but had decreased to less than 2% by 1900 (Lamagni et al., 2008). In the 1980s an unexplained wave of GAS infections occurred in Europe and North America, this time with remarkable localized tissue destruction and life-threatening systemic toxicity (Lamagni et al., 2008).

The most common site for GAS infection in humans in non-tropical regions is in the posterior oropharynx, particularly the lymphoid tissue which includes the tonsils (Watson et al., 2017). The immune function of the tonsils declines after puberty, which may explain why tonsillitis is rare in adults (Mayo Clinic, 2018). In outpatient medical visits for sore throat, GAS is diagnosed in about 20–40% of cases (Wessels, 2017). Routine clinical tests for GAS infection include throat swab tests for GAS antigens and blood sample tests for GAS antibodies (e.g., anti-streptolysin O).

The diagnosis of scarlet fever refers to streptococcal pharyngitis accompanied by a typical rash caused by a toxin (Wessels, 2017). Otitis media has been reported to involve GAS bacteria in only 3% of the cases (Segal et al., 2005). However, it seems to be the case that GAS infections causing otitis media were more common before penicillin was available, with a report of 50% of infected ears showing GAS (Valentine, 1924).

### Mechanisms of Autoimmunity

Autoimmunity occurs when the immune system acts against healthy tissues within the body. The *autoimmune mimicry hypothesis* states that antibodies generated from an immune response react with molecules in the tissues of the host, due to mimicry of bacterial and host antigens (Platt et al., 2017). This mechanism has been proposed to result in all secondary sequelae of GAS infections, including rheumatic fever and neurological disorders (Cutforth et al., 2016), though the exact mechanisms are not clear (Kothari, 2013). All humans produce antibodies or immune cells that are reactive with tissues of the body, which are normally eliminated before they result in damage. The development of autoimmune disorders appears to be related to the interaction between several different genes, involving both the production of self-reactive immune antibodies or cells and impaired elimination of these. It seems that a combination of several specific factors is necessary to trigger an autoimmune response to an infection (Cutforth et al., 2016).

### Neurological Autoimmunity in Relation to the Localization of the GAS Infection

In normal circumstances, the brain is protected by the blood-brain barrier, which should stop micro-organisms and proteins as well as immune cells or antibodies from passing into the brain. Within the nervous system, the microglia cells have an important role as immune cells. Based on a mouse model, Cutforth et al. (2016) proposed the hypothesis that T-cells, more specifically T_h_17 immune cells, play an important role in neurological autoimmune responses to GAS infections. According to this hypothesis, the lymphoid tissue in the tonsils of humans produces a large number of T_h_17 cells during GAS infections. The T_h_17 cells can pass the blood-brain barrier, for example to the olfactory bulb *via* the nasal tissue. The passing of the T_h_17 cells degrades the tight junctions of the blood-brain-barrier, allowing IgG antibodies and CD4^+^ T-cells to enter the brain. These antibodies and T-cells may in turn react with molecules within the brain tissue. Further, it is also hypothesized that viruses can support autoimmune responses by creating a pro-inflammatory state that “primes” the brain microglia to become overactive and to respond against brain tissue (Platt et al., 2017). This may be affected by the production of the pro-inflammatory cytokine TNF-α (Cutforth et al., 2016).

In conclusion, this model may explain the observation that GAS infections, particularly of the tonsils and the throat, have been reported to trigger autoimmune neurological sequelae, and that severe, recurrent, and combined infections may increase that risk.

### Rheumatic Fever

#### Rheumatic Fever (in Non-tropical Regions)

Rheumatic fever is assumed to be an autoimmune response caused by GAS infection, showing high heritability (Massell, 1997; Engel et al., 2011; Azevedo et al., 2012). Before the arrival of antibiotics, rheumatic fever was a relatively frequent and serious consequence of GAS in all populations, but it is now at a relatively low level in wealthy nations (Sika-Paotonu et al., 2017). The current incidence in the wealthy parts of the world is less than 10 annual cases per 100,000 population but has been reported to be up to 50 in developing parts of the world (Tibazarwa et al., 2008), though the numbers are likely to be uncertain. The symptoms may be severe, including rheumatic heart disease, polyarthritis, neurological symptoms such as Sydenham’s chorea (involuntary movements), subcutaneous nodules, and rash. It is estimated that rheumatic heart disease following GAS infections results in 233,000 deaths each year (Carapetis et al., 2005).

Rheumatic fever typically occurs from age 5 until puberty but may occur from about 3 to 19 years of age (Azevedo et al., 2012). In non-tropical parts of the world, the triggering infection typically has been a throat infection caused by *Streptococcus pyogenes*. It is estimated that there are over 600 million cases of GAS throat infections per year (Carapetis et al., 2005). Rheumatic fever is equally common in males and females (Sika-Paotonu et al., 2017). The symptoms typically begin about 2 weeks after the GAS infections (Sika-Paotonu et al., 2017), though sometimes may lag by several months (Swedo et al., 1998). The overt symptoms often resolve after 2 to 3 weeks, but sometimes with permanent damage to the heart valves.

The concept that some specific strains of GAS are “rheumatogenic,” i.e., causing autoimmune reactions such as rheumatic fever, has been a matter of debate. A recent review article and genetic study found that the classic rheumatogenic types of GAS were responsible for only 32% of the clinical cases of rheumatic fever (de Crombrugghe et al., 2020).

#### Rheumatic Fever in Indigenous Australian Children

It has been assumed that rheumatic fever is consistently caused by GAS pharyngitis, however, studies among indigenous Australian children indicates that this may not be the case in tropical regions. In this population, rheumatic fever is relatively common, despite evidence that sore throat and streptococcal pharyngitis show low rates, and that typical “rheumatogenic” strains of GAS are not present (McDonald et al., 2004, 2006). On the other hand, skin infection has been common, especially from GAS but also from other bacteria (McDonald et al., 2006). It has been proposed that the frequent rheumatic fever in this population may be caused by skin infections, and/or by other bacteria than *Streptococcus pyogenes* (Haidan et al., 2000; McDonald et al., 2004; Tanaka et al., 2008).

### Psoriasis

It has long been recognized that *guttate psoriasis* tends to be triggered by streptococcal pharyngitis, but more recently it has been proposed that the most common type, *plaque psoriasis*, is also a sequela of streptococcal infection (Allen and Miller, 2016). More specifically, it is argued that the development of plaque psoriasis requires the combination of a specific infection and a specific genetic profile of the host. The research data underlying this proposal is briefly reviewed in Presentation 2 in the Supplementary Material of this article.

### Neurological Symptoms From GAS Infections

#### Sydenham’s Chorea

Sydenham’s chorea is the most well described and well understood neurological disorder following GAS infections. The triggering infection, typically pharyngitis, may be minor and resolve without medical attention (Punukollu et al., 2016). Sydenham’s chorea typically occurs in prepubertal children but sometimes does occur in adolescents. The main symptoms are choreiform involuntary movements which can involve the whole body, and neuropsychiatric disturbances such as *obsessive-compulsive disorder* (OCD), hyperactivity, anxiety, emotional lability, irritability, inattention, and confusion (Cunningham and Cox, 2016; NORD, 2017). The symptoms of OCD can precede motor symptoms.

Sydenham’s chorea has long been considered an autoimmune disorder related to the basal ganglia. More recently it has been linked to antibodies against the dopamine receptor type D2 and described as a basal ganglia encephalitis. Dale et al. (2012) detected D2 antibodies in 10 out of 30 patients with Sydenham’s chorea but in none of the 40 controls (Fisher’s exact test, *p* < 0.0001). They found no subjects with D1 antibodies. Ben-Pazi et al. (2013) reported a correlation between the severity of the symptoms of Sydenham’s chorea and the ratio of the levels of D2/D1 antibodies. Non-motor symptoms showed the strongest correlation, *r* = 0.59, for aspects such as irritability, attention deficit, and hyperactivity. Motor symptoms showed a correlation of 0.47 with the D2/D1 ratio of antibodies. They interpreted the finding as suggestive for receptor imbalance resulting in increased sensitivity to dopamine signaling. This is in line with the observation that D2 antagonists can be used for the treatment of the symptoms of Sydenham’s chorea (Cunningham and Cox, 2016). There is a high incidence of movement disorders or emotional disorders in close relatives of patients with Sydenham’s chorea, suggesting an interaction between genetics and triggering factors (Punukollu et al., 2016).

#### PANDAS, PANS, and CANS

Following clinical observations of children showing OCD and tic disorders (Tourette syndrome), a group of researchers in 1998 proposed the definition of a subgroup of patients in neuropsychiatry. The acronym PANDAS was designated for the subgroup, for *Pediatric Autoimmune Neuropsychiatric Disorders Associated With Streptococcal Infections* (Swedo et al., 1998). Since the proposal of the concept of PANDAS, there has been an ongoing discussion as to what extent this constitutes a real diagnostic entity. For example, there is a significant overlap of the PANDAS diagnostic criteria and symptoms consistent with rheumatic fever. The main differences seem to be that rheumatic fever also includes chorea and other physical symptoms such as polyarthritis, carditis, subcutaneous nodules, and rash. To summarize, there appears to have been a general agreement that autoimmune disorders can result in sudden-onset neuropsychiatric disorders in children, but some authors have questioned the specific limitations of the diagnostic symptoms of PANDAS and the focus on streptococcal infections (Punukollu et al., 2016). For example, Singer et al. (2012) proposed the relatively wide concept of CANS, for *Childhood Acute Neuropsychiatric Symptoms*. CANS was intended to include various causes, and also a broader set of psychiatric symptoms, including OCD, general anxiety, phobias, developmental regression, poor concentration, emotional lability, and sleep problems, though with emphasis on OCD.

Swedo et al. (2012) modified the criteria for PANDAS and widened the concept to include other causes beyond GAS infection. The new concept was termed PANS, for *Paediatric Acute-onset Neuropsychiatric Syndromes*, with three main diagnostic criteria: (1) abrupt onset of OCD or severely restricted food intake; (2) concurrent abrupt onset of additional neuropsychiatric symptoms from at least two out of seven categories; and (3) symptoms not better explained by a known disorder, such as Sydenham’s chorea. The possible additional neuropsychiatric symptoms include anxiety, emotional lability, depression, irritability, aggression, behavioral regression, sensory or motor abnormalities, sleep disturbances, et cetera (Swedo et al., 2012).

The results from Dale et al. (2012) are suggestive of a difference in mechanism between PANDAS and Sydenham’s chorea. They detected antibodies against the dopamine D2 receptor in the sera of 10 out of 30 patients with Sydenham’s chorea (as mentioned above) but in none of 22 patients with PANDAS (nor the 40 control subjects). However, a common aspect of Sydenham’s chorea and PANDAS is the tendency of producing antibodies activating *calcium calmodulin protein kinase II* (CaMK II), which appears to result in neural excitation and increased transmission of dopamine (Chiarello et al., 2017). Overall the results from studies of antibodies and autoimmune responses in PANS/PANDAS are quite diverse, and the autoimmune basis of the symptoms has been a matter of debate (Chiarello et al., 2017). There are therefore no clear-cut criteria for the diagnosis of PANS/PANDAS. One test that is used for diagnosis of PANS/PANDAS is the “Cunningham Panel,” with five assays, including the CaMKII activity. The other assays are for antibodies against dopamine receptors D1 and D2, tubulin, and lysoganglioside (Cox et al., 2015; Cunningham et al., 2019). However, other researchers have argued that there is not enough evidence to limit the search for antibodies to only these five (Chiarello et al., 2017). Chiarello et al. (2017) proposed a clinical diagnostic pathway for PANS/PANDAS. A systematic review of the treatment of PANS/PANDAS is provided in Sigra et al. (2018). Treatment guidelines have been proposed by a consortium of clinicians and researchers, regarding anti-infection treatment (Cooperstock et al., 2017) and immunomodulation (Frankovich et al., 2017).

## Recent Case Reports of Stuttering in Children, Associated With GAS Infections

The author’s search of the more recent literature resulted in three case reports of children with stuttering associated with GAS infections, all three were boys.

### Six-Year-Old Male (Maguire et al., 2010)

The first case was reported by Maguire et al. (2010) and described a 6-year-old male with sudden onset of stuttering approximately 1 month after a streptococcal Group A throat infection had been diagnosed. The initial infection was documented with a rapid antigen test and involved sore throat, fever, and malaise as symptoms. The parents declined antibiotics at this time. The acute onset of stuttering 1 month later was characterized by sound and syllable repetitions, and silent blocking of speech. Four months from the initial infection the characteristic stuttering struggle behavior was developed, with facial grimaces and head twitches when stuttering occurred. At 5.5 months after the initial infection, the rapid antigen test identified the continued presence of GAS infection, and blood tests revealed high levels of GAS antibodies (antistreptolysin 0 and antideoxyribonuclease B). Treatment with penicillin (amoxicillin/clavulanic acid, 800 mg/day) was initiated, resulting in near resolution of the symptoms of stuttering within 2 weeks. At the time of the report 6 months later the patient remained free from symptoms of stuttering.

As far as to the knowledge of the current author know, Maguire et al. (2010) was the first to propose that an autoimmune condition may be at play in a subset of individuals who stutter. He proposed that such a reaction may be related to GAS infection and that it may be viewed as a symptom of PANDAS.

### Four-Year-Old Male (Lewin et al., 2011)

The second case, presented by Lewin et al. (2011), described a 4-year-old male, referred to as T. He was a mirror-image twin of P (T was left-handed, P right-handed). They were born somewhat prematurely at 32.5 weeks, with birth weight for T of 2.13 kg. Both brothers had a history of repeated confirmed throat GAS infections, most frequently around age 4, treated with antibiotics. The brother P did not stutter but showed symptoms of OCD, which was diagnosed as being part of a PANDAS syndrome. T showed throat clearing, which was considered a neuropsychiatric symptom, a tic. The repeated GAS infections were resolved by the removal of the tonsils and the adenoids for both brothers. T’s stuttering was described as notable for occurring when GAS tests were positive and improving with antibiotic treatment. Both the stuttering and the throat-clearing symptoms remitted following tonsillectomy and adenoidectomy, and the condition of T remained stable from that time.

### Nine-Year-Old Male (Ray et al., 2013)

The third case, reported by Ray et al. (2013), was a 9-year-old-male of premature birth at week 28, weight 0.75 kg. The neurological symptoms included stuttering, hyperactivity, and compulsive behaviors such as cleaning and not touching surfaces. He had a family history of autoimmune disorders, a mother with previous rheumatic fever and OCD, but no family history of stuttering. The boy was diagnosed with OCD, ADHD, and Tourette disorder. Further examination showed continuous high levels of GAS antigen (serum anti-streptolysin O), leading to consideration of a diagnosis of PANDAS. This diagnosis was supported by the observation of the increased severity of the neuropsychiatric symptoms with the pharyngotonsillitis and fever attacks. He did not respond to antibiotic treatment, but tonsillectomy normalized the serum anti-streptolysin O. Further examination led to the diagnosis of *Mevalonate Kinase Deficiency* (MKD), a genetic auto-inflammatory disease. This disease results in overactivation of the immune system, with periodic fever, musculoskeletal involvement, skin rashes, etc. Corticosteroid infusion was recommended during fever attacks, which his family declined. For the OCD symptoms treatment with sertraline 25 mg/day was initiated, titrated up to 50 mg/day. After 4 months the dose was reduced to 25 mg and stopped 3 weeks later. The symptoms of OCD had then been reduced to “mild.” Treatment of symptoms of ADHD was initiated, with atomoxetine 40 mg/day, with no observed side effects. At follow-up, there had been no severe recurrence of OCD symptoms, and the symptoms of ADHD were partially reduced. There was no explicit report regarding the outcome of the stuttering.

### Stuttering in Children With Early Antibiotic Treatment

It is of further interest to consider the report in Nabieva (2000). She studied 40 Russian children who stuttered, age 2.2–7 years. Of these children, 18 (45%) were reported to have been prescribed antibiotics during their first 3 years of life, and for 10 of the children during the first year. It was reported that in cases with antibiotics during the first year, the children typically stuttered when they began to talk. Children who had an initial period of fluent speech were said to usually have begun to stutter about 2 weeks after withdrawal of the antibiotics, or during 5 months after withdrawal. In the latter case, according to the report, the direct onset of stuttering tended to be associated with some minor emotional event, like having a nightmare or seeing a worm. It was proposed by Nabieva that antibiotics in itself may trigger stuttering. An alternative interpretation is that the children were treated with penicillin for a GAS infection, and the stuttering developed as an effect of the GAS infection. Possibly the antibiotic treatment was only partially successful, with renewed bacterial activity after the withdrawal of treatment. Alternatively, the treatment was successful but the infection had triggered a delayed autoimmune response.

The report by Nabieva does not include the typical frequency of antibiotic treatment before aged 3, or information on the type of infections treated. Still, the reports of the onset of stuttering some time after the withdrawal of antibiotics are of interest in relation to stuttering and GAS infection.

### Summary of Cases

In summary, the three cases showed confirmed GAS infections, repeated or extended over time. In all cases there were indications of a relationship between the GAS infection and the symptoms of stuttering: In two out of three cases the stuttering was improved or remitted by treatment with an antibiotic, but in one of these cases the GAS infection recurred until tonsillectomy and adenoidectomy, with lasting remittance of stuttering. In the third case, antibiotics were ineffective, but tonsillectomy showed a positive effect, and treatment with sertraline may have contributed to improving the symptoms of OCD. Two of the three cases showed symptoms of OCD or tics, covarying with the stuttering. It is of interest that the first case was reported to have an acute sudden onset of stuttering, without reports of other psychiatric or neurological signs. Yairi (2004) summarized that nearly 30% of the children in their studies showed a sudden onset of stuttering, that occurred over 1 day. The cause of these sudden onsets is unknown, but it seems pertinent to consider the possible influence of infections and autoimmune mechanisms. The reports of reduction of stuttering when the GAS infection was reduced indicate reversible neurological changes, rather than gross tissue damage.

## The Medical History of Stuttering Children in the 1930s

### Mildred Berry, Medical Record Data

It appears that the most thorough investigation of the medical history of children who stutter was carried out in the 1930s, by Dr. Mildred Freburg Berry, Rockford College, IL, USA (Berry, 1938). She stated that the motivation for this work came from a remark that was familiar to speech therapists at that time: “My son began to stutter immediately after a severe illness” (p. 97). Pediatric medical records of hospitals in Chicago were searched for data. Only records that appeared to be complete from infancy to 9–10 years of age were included in the analysis. The resulting cohort included a total of 430 children who stuttered and 462 matched controls. The mean age of onset of stuttering was 4.86 years. Diseases and medical events occurring before the onset of stuttering were compared with the information from the control group before age 5. Also, all later diseases and events up to 9 years of age were compared for the two groups.

One important strength of this study is that it is based on medical records, thereby avoiding the possible bias of retrospective recollection in surveys. Another strength is that events before and after the onset of stuttering are reported separately, until age 9. If the number of post-onset infections is normal it would indicate that the stuttering children were not especially susceptible to infection.

One potential weakness of this study is that the data registered at hospitals can be expected to underestimate the instances of minor disease, not requiring medical care. Therefore, the number of infections can be expected to be underestimated, for both groups.

#### Results From Berry (1938)

##### Infections More Frequent Before the Onset of Stuttering

Several infections were substantially more frequent in the stuttering group before the onset of stuttering (mean age 4.85 years), compared with control children before age 5, see Table 1. In contrast, the infections occurring after the onset of stuttering, and before age 9, did not differ from the controls in any remarkable way, see Table 2. The only disorder showing higher incidence after the onset of stuttering was rheumatic fever, with six cases in the stuttering group compared with none among the controls (*p* = 0.012, Fisher’s exact test). As an autoimmune sequala, rheumatic fever may occur several months after the GAS infection. Therefore, possibly the rheumatic fever was a sequela to the same GAS infection that triggered stuttering, but being diagnosed after the onset of stuttering.

**Table 1:** Summary of diseases in stuttering children before the onset of stuttering (mean age of onset 4.86 years) compared with control children before age 5, from Berry (1938).

	Stuttering			Controls	
Disease/symptom	*n*	*n* onset	%	*n*	%	Ratio St/C	Excess % stutt.	Fisher’s *p*
Total:	430			462
*Respiratory/neck infections with a strong link to neurological sequelae of GAS*:
Frequent severe tonsilitis:	70	2	16.3%	27	5.8%	2.8	10.4%	0.00001**
Scarlet fever (a complication of tonsillitis):	36	10	8.4%	20	4.3%	1.9	4.0%	0.013*
Scarlet fever, severe:	18		4.2%	4	0.9%	4.8	3.3%	0.0018**
Cervical adenitis with abscess:	19		4.4%	2	0.4%	10.2	4.0%	0.0001***
Frequent bronchitis:	67	6	15.6%	39	8.4%	1.8	7.1%	0.0012**
Respiratory infection with fever >=40.0C/104F:	27	3	6.3%	15	3.2%	1.9	3.0%	0.039*
*Other infections*:
Chickenpox:	89		20.7%	68	14.7%	1.4	6.0%	0.022*
Measles:	152	1	35.3%	144	31.2%	1.1	4.2%	0.20
Measles, severe:	9		2.1%	3	0.6%	3.2	1.5%	0.08
Pertussis/Whooping cough:		6	1.4%
Diphtheria:		4
Otitis media:	31		7.2%	64	13.9%	0.5	−6.6%	Rev: 0.0015**
*Autoimmune response to GAS infection*:
Rheumatic fever:	7	3	1.6%	3	0.6%	2.5	1.0%	0.21
*Conditions related to inflammation and immune response*:
Dermatitis/eczema:	24		5.6%	16	3.5%	1.6	2.1%	0.041^*
*Neurological conditions, possibly autoimmune response to infections*:
Encephalitis:	17		4.0%	2	0.4%	9.1	3.5%	0.0002***
Epilepsy:	12	6	2.8%	1	0.2%	12.9	2.6%	0.0012**
Convulsions:	24	6	5.6%	11	2.4%	2.3	3.2%	0.016*
*Other conditions, not more prevalent in the stuttering group before onset*:
Indigestion, intestinal:	14		3.3%	22	4.8%	0.7	−1.5%	Rev: 0.31
Malnutrition:	97		22.6%	151	32.7%	0.7	−10.1%	Rev: 0.0008***
Rickets (vitamin D deficiency):	96		22.3%	126	27.3%	0.8	−4.9%	Rev: 0.089

*p < 0.05, **p < 0.01, ***p < 0.001. “n onset” refers to the number of cases with onset of stuttering immediately upon the illness. “Ratio St/C” is the ratio of the incidences from both groups. “Excess % stutt.” is the number of percents that the incidence in the stuttering group exceeded the incidence of the control group. Fisher’s p is the p-value from Fisher Exact Test. Some conditions showed a lower incidence in the stuttering group compared with the controls. In these cases, the p-value is marked “Rev”. Rev, Reversed.

**Table 2:** Summary of diseases in stuttering children between the onset of stuttering (mean age of onset 4.86 years) and 9 years of age, compared with control children from age 5–9, from Berry (1938).

	Stuttering		Controls	
Disease/symptom	*n*	%	*n*	%	Ratio St/C	Diff. % stutt.	Fisher’s *p*
Total:	430	462
*Respiratory/neck infections with a strong link to neurological sequelae of GAS*:
Frequent severe tonsilitis:	20	5%	14	3.0%	1.5	1.6%	0.22
Scarlet fever:	19	4.4%	20	4.3%	1.0	0.1%	1.00
Cervical adenitis with abscess:	5	1.2%	5	1.1%	1.1	0.1%	1.00
Frequent bronchitis:	2	0%	3	0.6%	0.7	−0.2%	1.00
Respiratory infection with fever >=40.0C/104F:	15	3.5%	11	2.4%	1.5	1.1%	0.43
*Autoimmune response to GAS infection*
Rheumatic fever:	6	1.4%	0	0.0%	-	1.4%	0.012*
*Neurological conditions, possibly autoimmune response to infections*:
Encephalitis:	0	0.0%	3	0.6%	0.0	−0.6%	0.25
Epilepsy:	1	0.2%	2	0.4%	0.5	−0.2%	1.00
Convulsions:	1	0.2%	4	0.9%	0.3	−0.6%	0.37

*p < 0.05.

###### Frequent Severe Tonsillitis

The strongest statistical group difference was found for frequent severe tonsillitis, with *p* = 0.00001. Considering the “excess” number of cases in the stuttering group, this factor alone could explain 10.4% of the cases of stuttering. Tonsillitis can be caused by viruses or various bacteria, but frequent severe tonsillitis is typically caused by GAS infection. Before the onset of stuttering, frequent severe tonsillitis was 2.8 times more common in the children that would begin to stutter, while this condition was only 1.4 times more common in the stuttering children after the onset of stuttering. This supports the argument for the causal role of tonsillitis in the etiology of stuttering.

###### Scarlet Fever

The diagnosis of scarlet fever refers to tonsillitis caused by GAS, in combination with a typical rash. Scarlet fever was 1.9 times more common in the stuttering group before the onset of stuttering (*p* = 0.013). However, on the contrary, after the onset of stuttering scarlet fever was slightly more common in the control group. *Severe* scarlet fever showed an even stronger group difference before the onset of stuttering, with 18 vs. 4 cases (*p* = 0.0018).

###### Cervical Adenitis With Abscess

Cervical adenitis with an abscess is an inflammation with pus in a lymph node in the neck, typically caused by bacteria, for example, GAS. This condition occurred in 19 children who later began to stutter, compared with only two children in the control group. In contrast, after the onset of stuttering, there were equal numbers in both groups, five in each. This was the infection that showed the highest risk for stuttering as a sequala.

###### Frequent Bronchitis and High Fever

Bronchitis is not typically caused by GAS infection, but it does occur (Priftis et al., 2013). In the control group, 39 cases of frequent bronchitis were reported before age 5, compared with 67 cases among the children who stuttered, before the onset of stuttering. Somewhat surprisingly, after the onset of stuttering, only two in the stuttering group and three in the control group were reported. Respiratory infection with a fever over 40.0C/104F was also more common in the stuttering group, with a ratio of 1.9 before onset and 1.4 after.

###### Chickenpox and Measles

The rates of chickenpox (varicella) and measles were somewhat higher in the stuttering group compared with controls, before the onset of stuttering. These diseases have been very widespread among children, though typically without medical consultation. Neurological complications of chickenpox are considered to be relatively rare, with a rate of about one to three in 10,000 cases (Yılmaz and Çaksen, 2005). Typically, such neurological complications tend to affect structures outside the brain, such as the peripheral nervous system, the spinal cord, or the meninges (Yılmaz and Çaksen, 2005; Paul et al., 2010), though acute ataxia with antibodies to cerebellar and cerebral tissue has been reported after chickenpox (Adams et al., 2000). The incidence of neurological complications of measles has been estimated at 4 per 1,000 cases, often with encephalitis (Miller, 1964; Perry and Halsey, 2004).

Chickenpox before age 5 (approximately) was noted in the medical records for 20.7% of the stuttering group and 14.7% of the controls, and measles in 35.3% of the stuttering group and 31.2% of the controls. It is difficult to estimate the real incidences, but they can be expected to have been substantially higher since it is probable that most cases of these diseases did not result in medical consultation. One explanation for the slight group difference could be that the higher rate of severe respiratory infections in the stuttering group led to a higher rate of medical consultations, with a higher likelihood that chickenpox was also noted. There was only one report of measles occurring in direct relation to the onset of stuttering, and no such report for chickenpox. There are, however, nine reports of severe measles in the stuttering group before the onset of stuttering, compared with only three in the control group. It seems possible that a combination of measles and GAS infection can increase the risk of neurological sequelae.

Overall, the data does not provide any clear indication for a specific causal effect of chickenpox or measles on stuttering, though some contributing effects seem possible in the more severe cases with neurological complications.

##### Immune Disorders More Frequent Before the Onset of Stuttering

###### Rheumatic Fever

As mentioned above, rheumatic fever was remarkable in that it was the only condition that was substantially more common in the stuttering group both before and after the onset of stuttering, with seven vs. three cases before onset, and six vs. zero cases after the onset of stuttering. As mentioned previously, the symptoms of rheumatic fever may first appear several months after the triggering GAS infection, which may indicate that the stuttering and the rheumatic fever could arise from the same underlying infection. In total, 13 out of 430 stuttering children (3%) were diagnosed with rheumatic fever before age 9, compared with 3 out of 462 in the control group (0.6%).

###### Dermatitis/Eczema

Dermatitis/eczema are likely to be related to aberrant immune responses (Bos et al., 1992; McGirt and Beck, 2006), and atopic dermatitis has been proposed to be linked to an immune response triggered by *staphylococcal* bacteria (Gantz and B Allen, 2016). Dermatitis/eczema was more common in the stuttering group, with a ratio of 1.6, *p* = 0.041. An elevated rate of eczema in children with speech disorders was also reported by Strom and Silverberg (2016).

##### Neurological Disorders More Frequent Before the Onset of Stuttering

###### Encephalitis

Encephalitis, inflammation of the brain, can have many different causes. For example, childhood encephalitis may be the result of an autoimmune response triggered by infections (Barbagallo et al., 2017). Encephalitis can be global or localized to a specific structure, for example, some part of the basal ganglia (Dale et al., 2012). In the data from Berry (1938), encephalitis was related to stuttering, with 17 cases in the stuttering group before onset compared with two within the controls (*p* = 0.0002), After the onset of stuttering no cases of encephalitis were reported for the stuttering group, however, three cases were reported from the control group between ages 5 and 9 years.

###### Epilepsy and Convulsions

Epilepsy and convulsions are neurological symptoms, which also appear to be related to the onset of stuttering. Before the onset of stuttering, 12 children in the stuttering group were reported to have a diagnosis of epilepsy, and 24 had convulsions, compared with 1 and 11 in the control group (*p* = 0.0012 and 0.016, respectively). After the onset of stuttering, there were no indications of higher incidences in the stuttering group (*p* = 1.0 and 0.37).

##### Conditions Being Less Frequent Before the Onset of Stuttering

###### Nutrition and the Digestive System

The data on malnutrition indicated better status for the group of stuttering children compared with controls, with 22.6% cases of malnutrition in the stuttering group, compared with 32.7% among the controls. Using Fisher’s exact test this distribution results in *p* = 0.0008, implicating that it is unlikely to be a random effect. What could be the basis of this group difference? There is a physiological mechanism linking malnutrition to reduced risk for autoimmune attacks against the nervous system: the hormone *leptin*. Leptin is produced by fat tissues of the body (adipose tissue), and first became known as a molecule signaling satiety, decreasing hunger. With reduced energy intake the levels of leptin are lowered. It has later been shown that leptin also has an important and complex role as a regulator of immune responses. Low serum leptin concentration serves as a biomarker for malnutrition and is related to reduced generation of proinflammatory cytokines and increased risk for infectious disease (Maurya et al., 2018). On the other hand, higher levels of leptin are involved in a range of autoimmune diseases (La Cava, 2017). For example, experimental studies in mice indicate that leptin is required for the induction and maintenance of autoimmune responses within the brain (Matarese et al., 2001). The level of leptin typically become increased during infection and inflammation, and it has been shown in mice that starvation may prevent this increase in leptin and attenuate symptoms of autoimmune reactions (Sanna et al., 2003).

Based on the current findings regarding the role of leptin in autoimmunity it may be hypothesized that the magnitude of malnutrition among preschool children in Chicago during the 1930s was sufficient to result in some protection against autoimmune reactions. As a result, the children who did develop autoimmune reactions would tend to have a better average nutritional status.

###### Otitis Media

Surprisingly, the stuttering group was reported to only have half the incidence of otitis media, inflammation of the middle ear, compared with controls, *p* = 0.0015. As discussed above, otitis media is not typically caused by GAS infections, and ear infections appear to be less prone to generating autoimmune reactions compared with throat infections. It is, however, still surprising that the stuttering group showed substantially lower incidence. Could this be a real effect? Before antibiotics were available, chronic secretory otitis media could have a significant impact on hearing for extended periods, especially in the case of bilateral infection. In a more recent study, from Finland, 42% out of the 232 children with acute otitis media cases showed bilateral otitis (Uitti et al., 2013). A speculative proposal could be that impaired hearing associated with chronic otitis media had a protective effect on the risk to develop stuttering. The effect that masking of auditory input often improves stuttering is well documented (Dewar et al., 1976), though poorly understood. In Alm (2004), section 7.6, the relation between impaired hearing and decreased stuttering is reviewed and discussed.

#### Discussion of Results From Berry (1938)

In conclusion, the data from Berry (1938) indicates that the onset of stuttering was preceded by some type of GAS infection more frequently than would be expected based on the data from the control group. This is particularly the case for GAS *throat* infections, which is of particular relevance in this context. As discussed above section, throat infections of GAS have been linked to cerebral autoimmune reactions, such as Sydenham’s chorea and PANDAS.

### Severina Nelson, Comparing Familial and Non-familial Stuttering in the 1930s

Another large study of childhood stuttering in the 1930s was undertaken by Severina Nelson, Urbana, IL (Nelson, 1939). Her survey study focused on patterns of heredity but included some potentially relevant medical information. There were 204 stuttering propositi (i.e., the main persons of investigation), age 4–30 years. Information was collected from the propositi and their families.

An interesting aspect of the Nelson data is that she divided the propositi into two groups, one with stuttering in the family and one without. Analysis of this type of data could be expected to indicate the relative influence of genetic vs. non-genetic factors. If GAS infections play a causal role in stuttering it may be expected that the frequency and severity of GAS infections were higher in stuttering children without strong heredity for stuttering. However, a notable weakness of this study is that it is based on personal recollection, in some cases more than two decades after the onset of stuttering. There is a risk for recall bias, for example, that families with familial stuttering tend to attribute the stuttering to heritage while families without familial stuttering might tend to attribute the stuttering to certain events. Therefore, the results have to be interpreted as highly uncertain. A more detailed summary and discussion of this study can be found in Presentation 1 in the Supplementary Material of the current article.

A total of 104 stuttering propositi were considered to belong to families with familial stuttering, “stuttering families,” and 100 to “nonstuttering families” (though it is unlikely that there was a sharp distinction between these two categories). A main result in the current context was that one of the largest group differences was for severe respiratory infections, with approximately 31 instances reported in the “nonstuttering families” and approximately five instances reported in the “stuttering families.” This distribution results in *p* = 0.0000007 (Fisher’s exact test). This includes reports of tonsillectomy, scarlet fever, pneumonia, pertussis, “bad colds,” etc.

Though these data have to be considered as highly uncertain and approximate, they do follow the pattern from Berry (1938), supporting the hypothesis of GAS infections as an environmental factor with causal contributions for the onset of stuttering.

## The Parallel Decline of Rheumatic Fever and Stuttering Until the 1960s

### The Decline of Rheumatic Fever: Improved Conditions and Introduction of Penicillin

As discussed in above section, rheumatic fever is a systemic autoimmune disorder, sometimes with neurological symptoms. It is triggered by GAS infection, most often tonsillitis, and it typically occurs in children and young adolescents. The main hypothesis of the current article is that historically a major cause of childhood stuttering has been an autoimmune reaction resulting from GAS infections, particularly infections of the tonsils. In other words, this hypothesized mechanism causing stuttering is largely overlapping with the causal mechanism of rheumatic fever. If the hypothesis is correct it would be expected that the incidence of stuttering declined in parallel with the decline of the incidence of rheumatic fever. Furthermore, it can be expected that at least a part of this reduction is related to the introduction of penicillin for the treatment of tonsillitis. An expected difference between stuttering and rheumatic fever is that the relative reduction of the incidence of stuttering will be smaller because the GAS infection is likely to be a necessary factor in all cases of rheumatic fever, whereas stuttering often is unrelated to GAS infections.

**Figure 1:**
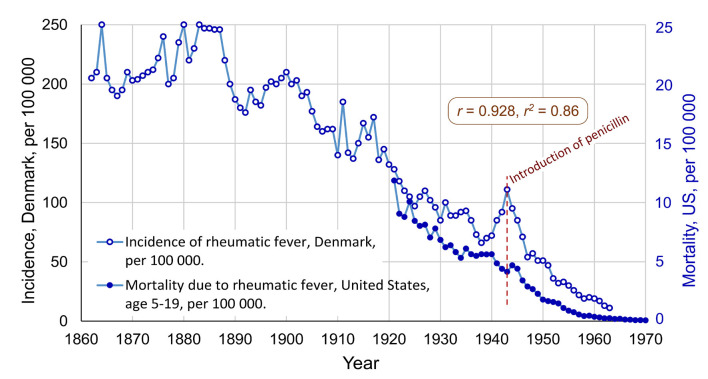
The international decline of rheumatic fever, with incidence data from Denmark (1862–1963) and mortality data from the United States (1921–1970). Correlation: *r* = 0.928, *r*^2^ = 0.81. Penicillin was introduced in 1943, in the US and in Denmark. Data for Denmark extracted from Figure 1 in Steer (2015), and data for the US extracted from Figure 4 in Massell et al. (1988). Both graphs changed from logarithmic to a linear scale (Note, the data for mortality before 1949 has been modified to attempt to compensate for a discontinuity in the data series related to change of the ICD criteria for diagnosis, see details in Data Sheet 1 in the Supplementary Material).

First, the decline of rheumatic fever will be discussed. Since the late 1800s, there has been a dramatic decrease in the incidence of rheumatic fever in the affluent parts of the world, despite the lack of vaccines against GAS infections. The changes appear to have been very similar in Western Europe and in North America. It would be expected that the incidence was reduced after the introduction of penicillin, but the decline began earlier, somewhat mysteriously (Gordis, 1985; Steer, 2015). Annual incidence data for rheumatic fever is available from Denmark, and mortality data from the US, see Figure 1.

In Figure 1 the decrease had already begun in 1888 and was almost linear until 1963. One important factor behind this decline appears to be improved conditions of living, especially when it comes to crowding in bedrooms (Kass, 1971; Bland, 1987). During World War I and II there was a problem of epidemics of rheumatic fever among young men in the military, who were living close to one another. Increases in the incidence in Denmark during these two wars can be seen in Figure 1. Another likely factor is that the virulence of the GAS bacteria changed so that the symptoms of GAS infections gradually became less severe (Lee and Wessels, 2006). Furthermore, there are proposals of dietary factors, in particular, that ingestion of meat and egg yolk may reduce the risk of the development of rheumatic fever (Massell, 1997, p. 220–221).

Penicillin was the first effective antibiotic against GAS tonsillitis (Plummer et al., 1945). At start, penicillin was restricted for military use, during World War II, but from 1943 it was supplied for civilian use both in the US and in Denmark. From 1944/1945 there was mass production in both countries, making it available in regular pharmacies (American Chemical Society, 1999; Skydsgaard, 2014).

There has been a debate regarding the role of penicillin in the decline of rheumatic fever. It would be reasonable to expect that penicillin had a major effect on the incidence of rheumatic fever, however, as shown in Figure 1, the decline began about 50 years earlier. As discussed above, this early decline appears to have been related to improved conditions of living. Though, Massell et al. (1988) showed that the number of severe consequences of rheumatic fever, such as carditis and deaths, was reduced at the time of the introduction of penicillin. Massell et al. (1988) argued that the effect of penicillin on the epidemiology of rheumatic fever started in about 1946^3^.

In summary, the incidence of rheumatic fever declined in Western Europe and in North America from about the start of the 1900s until early 1960, possibly with some acceleration of decline related to penicillin treatment from the mid 1940s.

### The Decline of the Incidence of Stuttering, Until the 1960s

#### The 1900s, Overall Perspective

The data regarding changes in the incidence/prevalence of stuttering are scarce, and the available data show great variation. Differences in criteria, methodology, and tradition have likely had a large impact on the results, making comparisons between studies difficult. It appears that the most thorough analysis of changes in the prevalence of stuttering in the United States during this period can be found in Dean and Brown (1977), primarily based on data from schools. These authors concluded that *“the prevalence of stuttering has steadily declined in the public schools between 190*4 a*nd the present”* (p. 162), but that *“there were no significant changes in incidence between 196*4 a*nd 1973”* (p. 165). It is striking that this pattern of change and timing almost exactly describes the pattern for rheumatic fever (see Figure 1), with a steady decline beginning in the late 1800s and reaching a floor level in the early 1960s.

The decline of the prevalence of stuttering can be exemplified by the reflections of Van Riper (1982), one of the most influential voices in the field of stuttering during the 1900s: *“When the author of this text began to practice in 1934, the high schools seemed full of stutterers and there were many adult stutterers to be encountered everywhere. This does not seem to be true today. We still have adult stutterers seeking our services but most of them come from afar”* (p. 49). Van Riper (1982) found this reduction to be supported by a report regarding the caseload of public school speech therapists in Illinois, USA, showing a reduction of stuttering from 7.4% of the total caseload in 1950–1951 to 3.2% in 1964–1965, i.e., a reduction of 57% in 14 years (Black, 1966, as cited by Van Riper, 1982, p. 49).

Van Horn (1966) reported the results of the examination of children in Kalamazoo, MI, USA, at 5 years of age. Yearly data were available for 1941–1944 and 1962–1965. The mean prevalence for the years in the early 1940s was 3.22% and for the early 1960s 2.47%, which represents a reduction of 23%. The author emphasized that the method for the examination had not changed during this period. It could be noted that these were preschool children, and the onset of stuttering as a result of GAS infection might well occur after 5 years of age, implying that this measure may underestimate the decrease of incidence as a result of GAS infection.

#### Annual Prevalence in Schools, 1945–1966: Introduction of Penicillin

##### Bivariate Correlation Analysis (Stuttering and Rheumatic Fever)

Penicillin was introduced to the public in 1943 both in the US and in Denmark. As discussed in above sections, the effect of penicillin on statistical measures of rheumatic fever began about 1946. If penicillin affected the incidence of stuttering it can be expected that this effect should be reflected in the prevalence data from schools during the following decades. Only one dataset with annual prevalence data of stuttering from the relevant period has been found by the author of this article. The data series is from grade 1–6 in Palo Alto schools, CA, USA from 1945 to 1966 (Jackson, 1967, as reproduced in Van Riper, 1982, p. 50)^4^.

**Figure 2:**
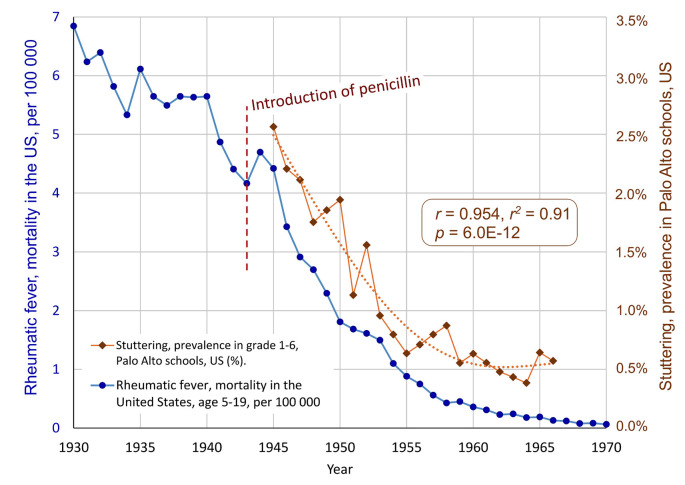
The parallel decline of stuttering and rheumatic fever, after the introduction of penicillin in 1943. The blue markers show the decline of the mortality due to rheumatic fever age 5–19, in the United States. The brown diamonds show the annual prevalence of stuttering in Palo Alto schools, California, grades 1–6 (Jackson, 1967, as reprinted in Van Riper, 1982). A polynomial trend line is fitted to the stuttering time series. The correlation is *r* = 0.954, *r*^2^ = 0.91, *p* = 6.0E-1 (Note, as mentioned in Figure 1, the data for mortality before 1949 has been modified. The correlation without the data before 1949 is *r* = 0.898 with *p* = 4.2E-07).

The data shows a dramatic decrease in the prevalence of stuttering in the Palo Alto schools, from about 2.56% in 1945 to a mean level of about 0.61% from 1955 to 1966, see Figure 2. The correlation between the school prevalence of stuttering and the mortality due to rheumatic fever for the overlapping period (1945–1966) is *r* = 0.954, *r*^2^ = 0.91, with *p* = 6.0E-12. This means that 91% of the variance of the mortality of rheumatic fever in the US after the introduction of penicillin is shared with the prevalence of stuttering in Palo Alto schools’ grades 1–6. The likelihood that this is a random effect is extremely low. The result supports the hypothesis of a link between GAS infections and stuttering.

**Figure 3:**
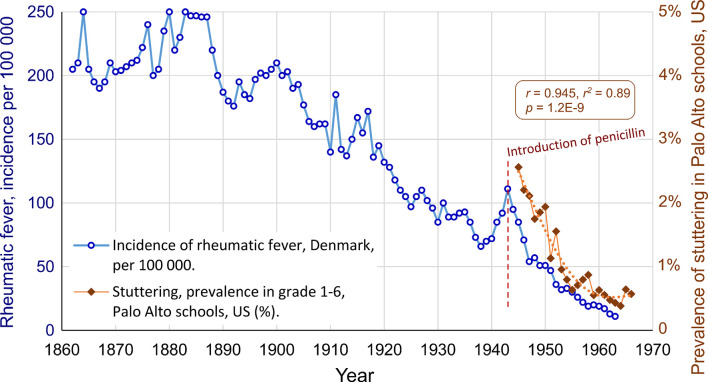
The parallel decline of stuttering and rheumatic fever in different continents, after the introduction of penicillin in 1943, in the US and in Denmark. The correlation is *r* = 0.945, *r*^2^ = 0.89, *p* = 1.2E-9.

The prevalence data for stuttering from Palo Alto can also be compared to the incidence data from Denmark, see Figure 3. The correlation is almost the same as with the mortality data from the US: *r* = 0.945, *r*^2^ = 0.89, *p* = 1.2E-9. This exceptionally high correlation between two seemingly unrelated conditions in two different continents suggests a major common factor. In this case, the major common factor is proposed to be the introduction of penicillin.

One limitation of the analysis is that I have only found annual prevalence data of stuttering from Palo Alto. Though, this data series does fit strikingly well with the overall US pattern summarized by Dean and Brown (1977) discussed above: decline until 1964, but no signs of decline between 1964 and 1973. Some specific examples of the decline of stuttering in public schools were highlighted by Van Riper and presented above. For example, data from Illinois showed a decline in the percentage of stuttering in the total caseload of public school speech therapists, from 7.4% in 1950–1951 to 3.2% in 1964–1965, which means 57% reduction (Black, 1966, as cited by Van Riper, 1982, p. 49). During the same period, the reported prevalence of stuttering in the Palo Alto schools was reduced by 50%, i.e., a similar rate of decline.

##### Multiple Regression, Controlling for Possible Confounding Variable

**Figure 4:**
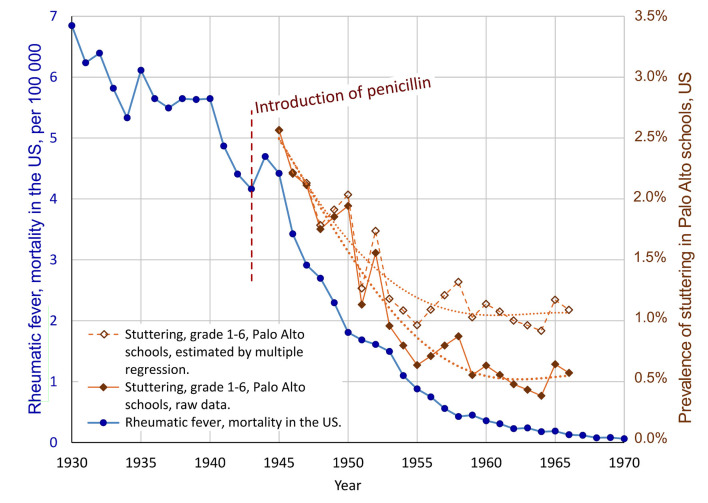
Result of multiple regression analysis of the possible confounding effect of the increasing number of students in Palo Alto schools, hypothetically resulting in reduced detection rate for stuttering. The unfilled diamonds show the stuttering data adjusted according to the multiple regression model, with the possible effect of an increasing number of students on the rate of detection of stuttering students removed, resulting in a more conservative estimate of a decline of stuttering. The correlation between the mortality due to rheumatic fever, and the prevalence of stuttering, according to the multiple regression, is *r* = 0.92, with *p* = 1E-9.

According to the data from Van Riper (1982), the number of children in Palo Alto grade 1–6 increased from 1,555 to 8,305 between 1945 and 1965. With this rapid growth in schools, it may be speculated that the detection rate for stuttering among the students was reduced for some reason. This means that the increase in students may be a confounding variable. To control for this possible confounder, and to make a more conservative analysis, multiple regression can be used: The dependent variable is the prevalence of stuttering in grade 1–6, Palo Alto schools (*StutteringPrevalence*). The two independent predictor variables are the annual mortality rate of rheumatic fever in the US (*RheumFever*) and the number of students in Palo Alto schools (*#Students*). This analysis results in *R*^2^ = 0.922 and *p* = 3E-11, implying that the model with *RheumFever* and *#Students* as predictors were able to account for 92.2% of the variance of *StutteringPrevalence* (software: Statistica 13). Though, only *RheumFever* showed statistical significance as predictor, with *p* = 0.0017 (partial correlation 0.64, standardized beta = 0.67, and beta = 2.8E-4). The variable *#Students* got *p* = 0.11 (partial correlation −0.36, and standardized beta = −0.31). The standardized beta values indicate that the influence of the predictor *RheumFever* showed more than double the influence of the variable *#Students*. Based on this regression model, the hypothesized influence of the increase in the number of students can be removed from the stuttering prevalence data. The resulting estimated prevalence series is plotted in Figure 4, unfilled diamonds. In this estimation, a relatively stable level of stuttering is reached from about 1959, with a mean level of 1.05% (*SD* = 0.087%) for the period 1959–1966.

In summary, the multiple regression analysis supports a relationship between the decline of rheumatic fever and the decline of stuttering, during the same period. Based on the analysis it seems possible that the dramatic reduction of the prevalence of stuttering in the Palo Alto schools was a combined effect of a real decline in the incidence and an artifact related to reduced detection rate following an increased number of students. Based on the standardized beta coefficients it may be estimated that about 68% of the reported reduction of stuttering may be attributed to a real decline, and about 32% to reduced detection rate as an effect of an increasing number of students. This would imply a reduction in prevalence from approximately 2.56% in 1945 to about 1.05% from 1959. Of course, the exact figures are very uncertain, and the initial 2.56% appears to be high in comparison with other studies.

#### Was there a Decline of Stuttering Caused by GAS Already From 1900 to 1945?

As discussed above, Dean and Brown (1977) reported that their compilation of data from the US, at a national level, indicated that the prevalence of stuttering had *“steadily declined”* (p. 162) from the early 1900s to the early 1960s. Further, as shown in Figure 2, the prevalence of stuttering appears to have followed the same trajectory as rheumatic fever between 1945 and the early 1960s. Does this mean that we can expect that the prevalence of stuttering followed the trajectory of rheumatic fever already from the early 1900s (as illustrated in Figure 3)? If this was the case, it would imply that the rate of stuttering was very high in the late 1800s. The compilation of data on stuttering from Dean and Brown does indicate a reduction of prevalence between the early 1900s and 1945, but we can not conclude that the magnitude of this reduction was the same for stuttering and rheumatic fever. The high correlation after 1945 suggested by Figure 2 is likely to have been caused by the specific effect of the introduction of penicillin on the frequency and severity of GAS tonsillitis. The decline of the incidence of rheumatic fever between 1900 and 1945 appears to be related to improved hygienic conditions, but also to poorly understood changes in the genetics of GAS strains. Possibly such genetic changes did not affect the risk for rheumatic fever and stuttering to the same degree. So, in conclusion, the available information does suggest some reduction of the incidence of stuttering caused by GAS infection between 1900 and 1945, but the correlation with the incidence of rheumatic fever is probably smaller compared with the period after the introduction of penicillin.

#### Estimation of the Size of the Decline of Stuttering Related to GAS

As discussed above, the historical prevalence data for stuttering is quite uncertain, for many reasons. Though, if the reasoning above is correct, is it possible to estimate the approximate size of the decline of the prevalence of stuttering in schools during the period 1900–1964? The raw data from Palo Alto suggests a very large decline, with about 75% reduction from 1945 to the late 1950s. If using the adjusted Palo Alto data from the multiple regression, the decline for this period would be about 59%. However, it is likely that the change in prevalence in Palo Alto was documented and published because of its striking magnitude. If there was a general decline of stuttering in US schools, the magnitude of the decline would have varied, with Palo Alto probably among the schools with the largest decline. Detailed analysis of national US data for the historical prevalence of stuttering is outside of the scope of this article but would be of interest.

## Discussion

### Summary and Evaluation of the Evidence for Stuttering Secondary to GAS Infection

The arguments for the hypothesis that untreated GAS infections may cause stuttering will be summarized and evaluated below^5^.

#### Medical Record Data From the Onset of Stuttering in the 1930s

The data from Berry (1938) provide unique information regarding medical conditions preceding the onset of stuttering, from a time before the introduction of penicillin. Rather than relying on personal recall, it is based on the hospital medical records of 892 children up to age 9 (430 children who stuttered and 462 non-stuttering controls). Only cases for which the medical record appeared to be complete until age 9 were included. For the children who stuttered the data is divided into events before and after the onset of stuttering (mean age 4.86 years). For the control group, the data is divided into events before and after age 5.

The single most significant diagnosis occurring before the onset of stuttering was frequent severe tonsillitis, affecting 16.3% (70 cases) of the stuttering children before the onset of stuttering, compared with 5.8% (27 cases) of the control group children before age 5 (*p* = 0.00001). The unexpected excess incidence of frequent severe tonsillitis in the stuttering group was 10.4% or 45 cases. This may represent the proportion of the children for which tonsillitis resulted in stuttering. It was mentioned above that Sydenham’s chorea may be triggered by minor GAS pharyngitis, resolving without medical attention (Punukollu et al., 2016). Considering that the data in Berry (1938) comes from hospital records, in the 1930s, it seems likely that the total number of GAS tonsillitis and pharyngitis are substantially underestimated.

There seems to be a delay in the onset of stuttering after tonsillitis: based on the data, it is estimated that tonsillitis triggered stuttering in 45 cases, still there are only two cases with onset of stuttering reported in direct association with tonsillitis. For rheumatic fever, the typical delay is around 2 weeks but may be several months. Long delays between triggering factors and expressed symptoms will obscure causal relations. In particular, this would be the case if GAS infections before the onset of speech may lead to stuttering later in speech development. The incidences for the various infections in Berry (1938) may refer to the same children, which means that it is not possible to calculate the number of children who had bacterial infections.

The other relevant infections before the onset of stuttering included cervical adenitis with abscess, which was approximately 10 times more frequent in the stuttering group, with 19 vs. 2 cases. Severe scarlet fever was more than four times more common, with 18 vs. 4 cases. Overall, it is the infectious diseases that stand out before the onset of stuttering, specifically the ones related to GAS infections. In contrast, *after* the onset of stuttering, the rate of GAS infections was almost identical in the two groups. This indicates that the high frequency of pre-onset infections in the stuttering group can not be explained as an effect of generally low levels of immunity among stuttering children. The pattern of strong group differences in the rate of GAS infections before the onset of stuttering, and equal rate after the onset of stuttering, strongly suggest a causal effect of GAS infections in relation to stuttering.

Two conditions showed significant group differences in the opposite direction, that is, lower in the stuttering group. That was otitis media (31 vs. 64, *p* = 0.0015) and malnutrition (97 vs. 151, *p* = 0.0008). As discussed above, these may be real effects, so that impairment of hearing and malnutrition could have paradoxical protective effects in the development of stuttering.

#### The Parallel Decline of Stuttering and Rheumatic Fever

The main hypothesis of this article was evaluated above by comparison of the available information regarding changes in the incidence of stuttering with changes in the incidence of rheumatic fever. The relevant data is scarce, but the available information does indicate striking parallels between the decline of rheumatic fever and stuttering, and a rapid decline in the incidence of stuttering after the introduction of penicillin. In the multiple regression model, the decline of rheumatic fever showed significance as a predictor for the decline of stuttering in the Palo Alto schools (*p* = 0.0017), while the increase of the number of students in the schools showed weaker predictive power (*p* = 0.11). Though, the data does not allow any detailed estimates of the size of the decline of the incidence of stuttering from the early 1900s to the early 1960s.

In the process of working with this article, the Danish data regarding rheumatic fever were found before the national data from the US. The correlation between the Danish data and the Palo Alto data for stuttering was extremely high: *r* = 0.945, see Figure 3. If this was a valid finding, with a causal connection, one would expect that national data on rheumatic fever from the US could get an even higher correlation, because of greater proximity to the students in Palo Alto and because a larger sample will reduce the random variations of the data. Indeed, the national US data on mortality due to rheumatic fever did show an even higher correlation: *r* = 0.954, providing strong evidence for a real connection.

It is a limitation that we only have one dataset with annual data for stuttering, from Palo Alto schools. This series was likely published because of the striking decline. This can be viewed as an example of selection bias, which limits our ability to generalize the size of the decline. However, it should here be emphasized that the correlation is independent of the absolute size of the decline; the correlation is based on the timing and the shape of the change. The extremely low *p*-values for the correlations (6.0E-12 for the mortality of rheumatic fever) indicates that, in all likelihood, there was a real decline of stuttering in parallel with the decline of rheumatic fever. Correlation does not in itself imply causation, but in this case, it seems difficult to conceive a strong link between variations in stuttering and rheumatic fever that does not involve the causal effects of GAS infections.

#### Reports of Recent Cases of Stuttering Following GAS Infections

Only three recent case reports of stuttering following GAS infections were found in this review. All three cases had confirmed pharyngeal GAS infections. In one case sudden onset of stuttering was reported approximately 1 month after the GAS infection. In all three cases, antibiotics or tonsillectomy were reported to be effective. The rapid reduction of stuttering in relation to a reduction of the GAS infections in some of these cases suggests that the neurological changes were reversible. These case reports are not conclusive, but they do suggest that stuttering secondary to GAS infections may still occur and that they may be treatable, at an early stage, with antistreptococcal interventions.

#### Possible Causal Mechanism

It is well established in the literature that untreated GAS infections may result in autoimmune reactions, including neurological symptoms in predisposed individuals. This is especially the case for pharyngeal GAS infections of the tonsils in prepubertal children. Studies indicate that the lymphoid tissue in the tonsils of humans produces a large number of T_h_17 cells during GAS infections, which are then able to pass and degrade the blood-brain barrier. This may allow IgG antibodies and CD4^+^ T-cells to enter the brain. The GAS bacteria produce a wide range of extracellular molecules. Some of them act as superantigens, resulting in a general activation of the immune system. Other molecules produced by the GAS bacteria may mimic molecules within the nervous system. This can result in the production of antibodies directed against specific neural structures. For example, both in Sydenham’s chorea and in PANDAS there is a tendency to produce antibodies against CaMK II, which appears to increase transmission of dopamine (Chiarello et al., 2017). Another example is the presence of antibodies against the dopamine D2 receptor in some patients with Sydenham’s chorea (Dale et al., 2012). There are also indications that simultaneous viral infections can support autoimmune responses by an increase of pro-inflammatory cytokines.

Possibly the risk for developing stuttering from GAS infections is related to individual factors such as age (higher risk in preschool age), sex (higher in boys), and genetics. The data from Berry (1938) indicates that impairment of hearing could have a protective effect against the development of stuttering and that malnutrition may reduce the risk of autoimmune reactions from GAS infections.

Sydenham’s chorea and PANDAS are examples of childhood neurological autoimmune reactions caused by GAS infections. These conditions are often accompanied by a range of symptoms with sudden onset, such as reduced eating (possibly as a result of increased leptin levels caused by the inflammation), OCD (e.g., fear of germs), elevated anxiety and emotional lability, hyperactivity and inattention, sleep disturbances, involuntary movements, and impairment of motor coordination (“clumsiness”). It is possible that some of these symptoms also tend to accompany stuttering triggered by GAS infection. It can be mentioned that studies of temperament and motor coordination of children who stutter have reported elevated scores for some of these aspects. This appears to be related to a minority of children who stutter; see for example Anderson et al. (2003), Eggers et al. (2010), and Alm (2014). If the existence of stuttering related to a GAS infection can be confirmed, should this be included as one of the symptoms of PANDAS? Current data does not indicate that stuttering is a frequent symptom of PANDAS. The comparison of symptoms and antibodies in Sydenham’s chorea vs. PANDAS suggests both overlap and differences. If stuttering occurs as a result of GAS infection it may be caused by a specific mechanism, differing from the mechanisms of PANDAS and Sydenham’s chorea. The author of this article would therefore advise against including stuttering related to GAS as a symptom of PANDAS, without more specific empirical support. Furthermore, by definition, stuttering should not be described as a (neuro)psychiatric disorder, but rather as a neurological symptom related to the initiation and control of speech movements.

Changes in the dopamine transmission appear to be an important aspect of the pathological mechanisms of neurological sequelae from GAS infections, such as Sydenham’s chorea and PANDAS. It is therefore of interest that the class of drugs that have shown the strongest effects on stuttering, making it better or worse, are the dopaminergic drugs (Alm, 2004; Maguire et al., 2004). The mechanism of GAS infection increasing the risk for stuttering may affect aspects of the dopamine system.

### Proposal of Conclusions

#### Evaluation of the Historical Evidence

Based on the review and analysis discussed above it is proposed that available data indicates that childhood GAS infection was a major cause of stuttering in North America, and probably in many other parts of the world, before antibiotic treatment of GAS was available to the public. The role of GAS infections as a cause of stuttering has likely differed substantially in different parts of the world, for example, related to the frequency of GAS tonsillitis, the availability of antibiotic treatment, and possibly also related to genetic differences.

The available historical data suggests a decline in the incidence of stuttering from the early 1900s to World War II and a more rapid decrease from 1945 to about 1960.

#### The Current Role of GAS Infections as a Cause of Stuttering

The magnitude of GAS infections as a causal factor for stuttering today is not known. Sudden onset of new symptoms appears to be a characteristic of neurological sequelae of GAS infections, such as PANDAS. According to Yairi (2004), nearly 30% of the children in their studies had a sudden onset of stuttering, occurring in a single day, typically without no apparent causal event. It would seem important that the possible link between stuttering and GAS infections is investigated further, in particular for children with sudden, unexplained onset of stuttering.

One method for gauging the current incidence might be to compare with the current incidence of rheumatic fever. This means that in populations with a current high incidence of rheumatic fever one could also expect a higher incidence of stuttering caused by GAS infections^6^. The multiple regression analysis above provides an indication of the incidence of stuttering in relation to the incidence of rheumatic fever. According to the data in Tibazarwa et al. (2008), an incidence of about five cases per 100,000 population may be expected in North America and Western Europe. According to the multiple regression analysis, five annual cases of rheumatic fever could be expected to correspond to about a 0.10% prevalence in school-age stuttering, secondary to GAS infection. If the overall prevalence of school-age stuttering is 1%, then approximately one out of 10 school-age children who stutter would have stuttering related to GAS infection. However, this estimation is very uncertain.

### Proposals for Research

#### Historical Data

If it is possible to retrieve further historical time series data regarding the prevalence of childhood stuttering it would elucidate the hypotheses proposed in this article. Data from the entire 1900s is of relevance, though with particular focus on the decades after the introduction of penicillin.

#### The Current Situation, Childhood Stuttering

Very little is known regarding the possible current relevance of streptococcal infections as a cause of childhood stuttering. There are very few current cases reported in the literature, though these cases do indicate that such a mechanism exists. The first step of the continued research could be to search for more cases, through clinical investigation, and attempt to treat confirmed cases of GAS infection^7^. If possible, the involvement of local expertise on PANS/PANDAS may be fruitful, utilizing routines for PANS/PANDAS assessment. Proposals for treatment guidelines for PANS/PANDAS has been published by Cooperstock et al. (2017) for anti-infection treatment and by Frankovich et al. (2017) for immunomodulatory therapy. A preliminary screening form for signs of stuttering related to GAS infections is included in Table 1 in the Supplementary Material of the current article. For children who score highly on this screening, it may be relevant to proceed with a routine clinical investigation regarding signs of GAS infection. This could include, for example, the standard throat swab tests for GAS antigen, blood test of antistreptolysin antibodies, and CRP test of inflammation. If signs of GAS infection are found, the standard clinical treatment of GAS infection may be applied. If the stuttering and other symptoms are improved it would support the hypothesized link. In the case of partial improvement or relapse, with continued indications of infection, further medical treatment measures may need to be considered. It would also be of great interest to get antibody data, for example from the “Cunningham Panel,” including the CaMKII activity.

It seems important to gather data from cases globally and within different populations since the spectrum of infections, bacterial strains, and autoimmune responses differ. If the existence of current cases of stuttering linked to GAS infections can be confirmed, more structured studies can be initiated. This might include using assays for antibodies linked to PANS/PANDAS and Sydenham’s chorea, and genetic studies of affected persons.

## Data Availability Statement

All datasets presented in this study are included in Data Sheet 1 in the Supplementary Material.

## Author Contributions

The author confirms being the sole contributor of this work and has approved it for publication.

## Conflict of Interest

The author declares that the research was conducted in the absence of any commercial or financial relationships that could be construed as a potential conflict of interest.

## Footnotes

^1^The result of this study of brain FDOPA uptake is of potential great interest, but because of uncertainties of the result there is a need for replication before it can be interpreted. The uncertainty is based on small number of participants (three stuttering), control group scanned earlier, and unexpectedly large group difference.

^2^It has been shown, however, that also strains of *Streptococcus dysgalactiae equisimilis* can possess Group A antigen, though these bacteria seems to be relatively rare in human infections (Tanaka et al., 2008).

^3^The book by Massell (1997) can be strongly recommended for anyone interested to learn about the history of rheumatic fever and the history of treatment of streptococcal infections.

^4^The current author has not been able to retrieve the original report, by R. M. Jackson, presented 1967 at a convention for the California Speech and Hearing Association. The graphs are reproduced in Van Riper (1982) page 50. The data from the graphs has been estimated by measurement in graphic software, and is included in Data Sheet 1 in the Supplementary Material.

^5^Some alternative interpretations of the reported decline of stuttering is discussed in the Supplementary Material (because of word limit for the article).

^6^However, this is not necessarily the case. In the review above it was discussed that neurological symptoms from GAS infections seems to be a particular effect of tonsillitis, while it is suspected that rheumatic fever in indigenous Australian children is typically an effect of skin infections. This type of rheumatic fever may show weaker association with neurological symptoms such as stuttering.

^7^An information sheet on PANDAS/PANS intended for Speech-Language Pathologists can be downloaded from www.mnsu.edu/comdis/kuster/related/pandas/pandashandout.pdf.

## Abbreviations

CaMK II: calcium calmodulin protein kinase II
CANS: Childhood Acute Neuropsychiatric Symptoms
GAS: Group A beta-hemolytic bacteria
OCD: obsessive-compulsive disorder
PANS: Paediatric Acute-onset Neuropsychiatric Syndromes
PANDAS: Pediatric Autoimmune Neuropsychiatric Disorders Associated with Streptococcal infections.
