# Supplementary material for: Streptococcal Infection as a Major Historical Cause of Stuttering: Data, Mechanisms, and Current Importance
Source: Front Hum Neurosci. 2020 Nov 9;14:569519. doi: 10.3389/fnhum.2020.569519 (PMC7693426; doi:10.3389/fnhum.2020.569519)
Supplement: Supplementary file 2 [file Presentation_1.pdf]

File: *Presentation 1.pdf*, supplemental material to *Alm, PA (2020). Streptococcal infection as a major historical cause of stuttering: data, mechanisms, and current importance. Frontiers in Human Neuroscience.*

frontiers

Supplementary Material

Data and discussion of study of stuttering from 1939, by Severina Nelson: comparing familial and non-familial stuttering

Nelson, S. E. (1939). The role of heredity in stuttering. J. Pediatr. 14, 642–654.

June 2, 2020

1 Retrospective survey data

A large study of childhood stuttering in the 1930s was undertaken by Severina Nelson, Urbana, Ill. (Nelson, 1939). Her survey study was primarily focused on patterns of heredity, but also included some medical information. There were 204 stuttering propositi (i.e. the main persons of investigation), age 4 to 30 years. Information was collected from the propositi and their families. A weakness of this study is that it is based on personal recollection, in some cases more than two decades after the onset of stuttering. This may explain the lower incidence of infections reported, compared to those in Berry (1939), which was based on medical records. However, an interesting aspect of the Nelson data is that she divided the propositi into two groups, one with stuttering in the family and one without. This can be expected to indicate the relative influence of genetic versus non-genetic factors. If GAS infections play a causal role for stuttering it may be expected that the frequency and severity of GAS infections were higher in stuttering children without strong heredity for stuttering. A total of 104 stuttering propositi were considered to belong to "stuttering strains" and 100 to "nonstuttering strains", though it is unlikely that there was a sharp distinction between these two categories.

An interesting aspect of this article is that the individual factors that the family associated with the onset of stuttering were described in words. For example: " In bed three months with infection of lymphatic glands; then frightened by dog, amnesia spells, highly strung disposition." A factor that requires consideration when evaluating this type of retrospective survey data is the risk for recall bias. In cases where there is familial history of stuttering, the heritage may be the preferred explanation for family, with the risk that other factors relating to childhood events are overlooked or underreported. In contrast, it is possible that cases without a familial history of stuttering might be more likely to attributed to specific childhood events. Further, when trying to summarize the information from the report some inconsistencies appear. Therefore, the summary below and in table 1 should be viewed as an approximate attempt to compile the diverse information from the report.

2 Results, familial versus non-familial stuttering

In total, 157 possible contributing factors were reported for the 100 propositi of the "nonstuttering strains", to be compared to 63 for the "stuttering strains". This supports the hypothesis of infections and other environmental "events" having a causal role for the development of stuttering, because the events are more frequent in stuttering persons with lower genetic predisposition for stuttering. This is in line with more recent data showing a similar pattern, see Poulos and Webster (1991) and Alm and Risberg (2007).

2.1 Severe respiratory infections

In accord with the results from Berry (1938) one of the strongest association was found for severe respiratory infections (see Table 1), with with 31 versus 6 reported instances, resulting in p = 0.00009. This includes reports of tonsillectomy, scarlet fever, pneumonia, pertussis, "bad colds", etc.

Table 1. Summary of approximate number of instances of diseases in a retrospective survey study from Nelson (1939). The children are grouped based on the occurrence of stuttering in their family and relatives, in "non-stuttering strains" and "stuttering strains". The numbers are the summarized estimated results from analyses of case comments (by the author of this article) published by Nelson (1939), and the statistics presented in the same article.

Disease/symptom	Non-stuttering strains		Stuttering strains		Fisher's *p*
	n	%	*n*	%	
Total:	100		104		
Neurological/psychiatric symptoms:					
Convulsions:	7	7%	2	2%	0.096
Sydenham's chorea (GAS infection):	3	3%	1	1%	0.36
"Nervous dispositions":	16	16%	6	6%	0.023
Respiratory infections, and related surgery:					
Scarlet fever (GAS infection with tonsillitis and rash):	8	8%	4	4%	0.24
Severe respiratory infections:	31	31%	5	5%	0.0000007
Tonsillectomy (probably because of repeated GAS tonsillitis):	5	5%	2	2%	0.27
Other disorders:					
Pertussis (whopping cough):	17	17%	9	9%	0.093
Hyperthyroidism:	3	3%	0	0%	0.12

2.2 Anxiety, possibly comorbid with Sydenham's chorea

The second strongest group difference in Table 1 is for "Nervous dispositions", with $p = 0.023$. For example, one of these cases was described as "Whooping cough, complications, chorea, extremely nervous". Childhood chorea in the 1930s is likely to have been Sydenham's chorea. As discussed in the main article, Sydenham's chorea is caused by GAS infection, and the patients often show various symptoms, including OCD, anxiety, and emotional lability. This is similar to PANS/PANDAS. It seems possible that at least some of the pediatric cases of stuttering in Nelson (1939) showing "extreme nervousness" did this as part of neuropsychiatric symptoms caused by autoimmune reaction from infections, i.e. Sydenham's chorea or PANS/PANDAS. In other words, what is reported as "Nervous disposition" may well reflect a sequela of GAS infection.

2.3 Pertussis (whooping cough)

The third strongest finding in Table 1 is the higher frequency of reports of pertussis in association with the onset of stuttering in the "non-stuttering strain", with 17% incidence compared with 9% incidence in "stuttering strain". Pertussis is a contagious bacterial disease. The typical symptom of pertussis is violent and rapid coughing, until there is no air in the lungs. The coughing can last for 10 weeks or more. In babies the cough can be minimal, and may present as apnea, with pauses of breathing (CDC, Centers for Disease Control and Prevention, 2017). It may result in serious and even fatal complications in babies and young children. The complications include pneumonia, convulsions, apnea, and encephalopathy. Early treatment with antibiotics can reduce the severity of the symptoms (CDC, 2017).

The exact childhood incidence of pertussis in the US in the 1930s is difficult to establish, but based on available figures the reported rate of 17% incidence pertussis in the "non-stuttering strain" of Nelson (1939) does not seem remarkable.[1] What makes it suspect in this context is primarily the difference between the "non-stuttering strain" and the "non-stuttering strain", with p = 0.093 for group difference, and that the families had associated the onset of stuttering with the pertussis. Childhood pertussis can result in neurological complications, believed to be caused by cerebral hypoxia, bleeding, or meningoencephalitis (Greenberg et al., 2005). Surprisingly, based on animal studies it has been proposed that the pertussis bacteria might have a protective effect against the development of autoimmune diseases (Lehmann and Ben-Nun, 1992). As a result of immunization programs in most countries, and use of antibiotics, the incidence of pertussis has declined substantially from the early 1940s to the 1960s.[2] In conclusion, the available information seems to indicate that pertussis has not had any specific or major effect in increasing the risk for stuttering.

---

[1] In the 1930:s in the United States the incidence was about 200,000 cases per year (CDC, Centers for Disease Control and Prevention, 2002). It has primarily been a disease of the childhood, with US data from Luttinger (1916) showing that about 80% of all patients were under 5 years of age, and only about 2% were over 15. The yearly birth rate in the US 1930s was about 600,000 (Bureau of the Census, 1939). If assuming that only children up to 10 years were affected this would mean that roughly about 33% of children had pertussis before age 10.

[2] In the United States about 8000 cases per year were reported around year 2000, but increasing (CDC, Centers for Disease Control and Prevention, 2002). Considering the increase in childhood population compared to the 1930s the current risk for childhood pertussis is about 1% compared to the risk in the 1930s.

2.4 Hyperthyroidism

In Table 1 there are 3 cases of hyperthyroidism out of the 100 cases in the "non-stuttering strain" compared to none in the "stuttering strain". This suggests the possibility of hyperthyroidism increasing the risk for stuttering. The typical incidence of childhood hyperthyroidism appears uncertain, but seems to have been substantially lower than 3%, both historically and more recently (Dinsmore, 1932; Robinson, 1940; McGrogan et al., 2008). The most frequent cause of childhood hyperthyroidism, though rare, is Grave's disease, which is an autoimmune disorder with production of antibodies that stimulates the thyroid gland to produce excess thyroid hormone (Léger and Carel, 2013). The incidence of Grave's disease in children is reported to be less than 3 per 100 000 person years, but more frequent in families with other autoimmune disorders. Hyperthyroidism may result in psychiatric symptoms, such as mood and anxiety disorders, and cognitive dysfunction (Bunevicius and Prange, 2006).

Considering the low general incidence of hyperthyroidism, the report of 3 cases among the children without familial stuttering is unexpected. To my knowledge, stuttering has not been reported as a symptom of hyperthyroidism in the literature. Glaser (1936), though, reported the experiences from two endocrinologists that childhood patients receiving high dosage thyroid treatment may show stuttering, or worsening of existing stuttering. Whatever the case, overall, it is expected that hyperthyroidism would have a marginal effect on the incidence of stuttering.

3 References

Alm, P. A., and Risberg, J. (2007). Stuttering in adults: The acoustic startle response, temperamental traits, and biological factors. *J.Commun.Disord.* 40, 1–41. doi:10.1016/j.jcomdis.2006.04.001.

Berry, M. F. (1938). A study of the medical history of stuttering children. *Speech monographs* 5, 97–114. doi:10.1080/03637753809374854.

Bunevicius, R., and Prange, A. J. (2006). Psychiatric manifestations of Graves' hyperthyroidism. *CNS drugs* 20, 897–909. doi:10.2165/00023210-200620110-00003.

Bureau of the Census (1939). Vital statistics of the United States 1937, part 1. Available at: http://data.nber.org/vital-stats-books/VSUS_1937_1.CV.pdf.

CDC, Centers for Disease Control and Prevention (2002). Pertussis--United States, 1997-2000. *MMWR. Morbidity and mortality weekly report* 51, 73.

CDC, Centers for Disease Control and Prevention (2017). Pertussis (Whooping Cough). Available at: https://www.cdc.gov/pertussis/about/index.html [Accessed February 18, 2020].

Dinsmore, R. S. (1932). Hyperthyroidism in Children: A Review of Fifty-Seven Cases. *Journal of the American Medical Association* 99, 636–638. doi:10.1001/jama.1932.02740600028007.

Glaser, E. M. (1936). Possible relationship between stuttering and endocrine malfunctioning. *Journal of Speech Disorders* 1, 81–89. doi:10.1044/jshd.0103.81.

Greenberg, D. P., von König, C.-H. W., and Heininger, U. (2005). Health burden of pertussis in infants and children. *The Pediatric infectious disease journal* 24, S39–S43. doi:10.1097/01.inf.0000160911.65632.e1.

Léger, J., and Carel, J. C. (2013). Hyperthyroidism in Childhood: Causes, when and how to treat. *Jcrpe* 5, 50–56. doi:10.4274/jcrpe.854.

Lehmann, D., and Ben-Nun, A. (1992). Bacterial agents protect against autoimmune disease. I. Mice pre-exposed to Bordetella pertussis or Mycobacterium tuberculosis are highly refractory to induction of experimental autoimmune encephalomyelitis. *Journal of Autoimmunity* 5, 675–690. doi:10.1016/0896-8411(92)90185-S.

Luttinger, P. (1916). The epidemiology of pertussis. *American Journal of Diseases of Children* 12, 290–315. doi:10.1001/archpedi.1916.04110150088007.

McGrogan, A., Seaman, H. E., Wright, J. W., and De Vries, C. S. (2008). The incidence of autoimmune thyroid disease: a systematic review of the literature. *Clinical endocrinology* 69, 687–696. doi:10.1111/j.1365-2265.2008.03338.x.

Nelson, S. E. (1939). The role of heredity in stuttering. *The Journal of Pediatrics* 14, 642–654.

Poulos, M. G., and Webster, W. G. (1991). Family history as a basis for subgrouping people who stutter. *J.Speech Hear.Res.* 34, 5–10. doi:10.1044/jshr.3401.05.

Robinson, S. K. (1940). The age distribution and sex incidence of hyperthyroidism. *Endocrinology* 26, 409–417. doi:10.1210/endo-26-3-409.
