# Supplementary material for: Streptococcal Infection as a Major Historical Cause of Stuttering: Data, Mechanisms, and Current Importance
Source: Front Hum Neurosci. 2020 Nov 9;14:569519. doi: 10.3389/fnhum.2020.569519 (PMC7693426; doi:10.3389/fnhum.2020.569519)
Supplement: Supplementary file 3 [file Presentation_2.pdf]

File: *Presentation 2.pdf*, supplemental material to *Alm, PA (2020). Streptococcal infection as a major historical cause of stuttering: data, mechanisms, and current importance. Frontiers in Human Neuroscience.*

frontiers

Supplementary Material

About psoriasis in relation to group A beta-hemolytic streptococcus (GAS)

August 12, 2020, Per Alm, per.alm@neuro.uu.se

It has long been recognized that *guttate psoriasis* tend to be triggered by streptococcal pharyngitis, but more recently it has been proposed that the most common type, *plaque psoriasis*, is also a sequela of streptococcal infection (Allen and Miller, 2016). More specifically, it is argued that the development of plaque psoriasis requires the combination of a specific infection and a specific genetic profile of the host.

Throat infections tend to be associated with exacerbation of plaque psoriasis, especially in cases with confirmed streptococcal infections (Thorleifsdottir et al., 2014). Though, the existence of GAS infections in persons with plaque psoriasis has not been clearly confirmed. In a study from Korea 11 out of 19 children with plaque psoriasis were reported to show high titers of antistreptolysin O, indicating GAS infection, as were 6 out of 18 adults (Kim et al., 2010). El-Rachkidy et al. (2007) reported that adults with plaque psoriasis did not differ from controls regarding antistreptolysin O, but that the summarized level of proteins secreted by *Streptococcous pyogenes* was significantly higher in the patients compared with healthy controls (effect size *Glass* Δ = 1.05). Allen et al. (2018) proposed that tonsil biofilms can isolate GAS bacteria, and therefore are not detected by swab tests or by blood antigen tests. This group of researchers reported the presence of biofilms in the tonsils of all of 10 patients with plaque psoriasis, and that the findings were similar to the findings from patients with chronic tonsillitis. However, specimens from healthy controls were not available for comparison.

There are several reports of attempts to treat plaque psoriasis using antistreptococcal interventions, though, according to the Cochrane review by Dupire et al. (2019), mostly low-quality studies. The results summarized here therefore need to be characterized as highly preliminary in need for further confirmation. A randomized control trial of tonsillectomy in patients with streptococcal-associated exacerbation of psoriasis resulted in no change in the control group, but with 13 out of 15 patients in the tonsillectomy group showing on average more than 50% improvement of the severity scores of the psoriasis (Thorleifsdottir et al., 2012). The improvement showed a correlation of 0.59 with the frequency of skin-homing T-cells. Saxena and Dogra (2005) claimed an average of 95% reduction of the severity scores for 30 patients with plaque psoriasis, after 48 weeks of treatment with penicillin injections (given fortnightly for 24 weeks and thereafter once a month). In a later, single-blind randomized trial, 28 patients were treated with oral azithromycin (Saxena and Dogra 2010), which can also reach intracellular bacteria. The treatment regime was 48 weeks with 500 mg daily for four days, followed by a gap of ten days. At the end of the study the control group showed no change,

while the treated group was reported to show an overall mean of 83% reduction of the psoriasis severity scores.

In summary, further studies of the possible relationship between plaque psoriasis and GAS infections are required. If such a link can be confirmed it would provide an interesting example of chronic GAS infections resulting in autoimmune symptoms, which may be of relevance for the further understanding of the neurological sequelae of GAS.

References

Allen, H. B., Jadeja, S., Allawh, R. M., and Goyal, K. (2018). Psoriasis, chronic tonsillitis, and biofilms: Tonsillar pathologic findings supporting a microbial hypothesis. *Ear, Nose & Throat Journal* 97, 79–82. doi:10.1177/014556131809700309.

Allen, H. B., and Miller, B. (2016). Psoriasis: A Sequela of Streptococcal Infection Similar to Acute Rheumatic Fever. *Clin Microbiol* 05. doi:10.4172/2327-5073.1000244.

Dupire, G., Droitcourt, C., Hughes, C., and Le Cleach, L. (2019). Antistreptococcal interventions for guttate and chronic plaque psoriasis. *Cochrane Database of Systematic Reviews*. doi:10.1002/14651858.CD011571.pub2.

El-Rachkidy, R. G., Hales, J. M., Freestone, P. P. E., Young, H. S., Griffiths, C. E. M., and Camp, R. D. R. (2007). Increased Blood Levels of IgG Reactive with Secreted Streptococcus pyogenes Proteins in Chronic Plaque Psoriasis. *Journal of Investigative Dermatology* 127, 1337–1342. doi:10.1038/sj.jid.5700744.

Kim, S. K., Kang, H. Y., Kim, Y. C., and Lee, E.-S. (2010). Clinical comparison of psoriasis in Korean adults and children: correlation with serum anti-streptolysin O titers. *Archives of dermatological research* 302, 295–299. doi:10.1007/s00403-009-1025-8.

Saxena, V., and Dogra, J. (2005). Long-term use of penicillin for the treatment of chronic plaque psoriasis. *European Journal of Dermatology* 15, 359–362.

Thorleifsdottir, R., Eysteinsdóttir, J., Olafsson, J., Sigurdsson, M., Johnston, A., Valdimarsson, H., et al. (2014). Throat Infections are Associated with Exacerbation in a Substantial Proportion of Patients with Chronic Plaque Psoriasis. *Acta Derm Venerol*, 0. doi:10.2340/00015555-2408.

Thorleifsdottir, R. H., Sigurdardottir, S. L., Sigurgeirsson, B., Olafsson, J. H., Sigurdsson, M. I., Petersen, H., et al. (2012). Improvement of psoriasis after tonsillectomy is associated with a decrease in the frequency of circulating T cells that recognize streptococcal determinants and homologous skin determinants. *The Journal of Immunology* 188, 5160–5165. doi:10.4049/jimmunol.1102834.
