# Supplementary material for: Streptococcal Infection as a Major Historical Cause of Stuttering: Data, Mechanisms, and Current Importance
Source: Front Hum Neurosci. 2020 Nov 9;14:569519. doi: 10.3389/fnhum.2020.569519 (PMC7693426; doi:10.3389/fnhum.2020.569519)
Supplement: Supplementary file 4 [file Presentation_3.pdf]

File: *Presentation 3.pdf*, supplemental material to *Alm, PA (2020). Streptococcal infection as a major historical cause of stuttering: data, mechanisms, and current importance. Frontiers in Human Neuroscience.*

Supplementary Material

Discussion, alternative explanations for the reported decline of stuttering?

August 17, 2020, Per Alm, per.alm@neuro.uu.se

Van Riper (1982) suggested that *"it is quite possible ... that if the incidence of stuttering has declined it may be due to the marked changes in the attitude of the society towards the stutterer, and to the parent and teacher education contributed by the public school clinicians"* (p. 49). One factor affecting the attitudes in the society was the 'diagnosogenic theory' by Wendell Johnson, introduced in 1942 (Bloodstein and Bernstein-Ratner, 2008). It had great influence on the attitudes towards stuttering in the 1950s and 1960s, but also later. One advice to parents based on the theory was to ignore early signs of stuttering. Theoretically, it might be hypothesized that this advice reduced the incidence of stuttering, or that it changed the diagnostic criteria and reports from public schools. In summary, there seems to be two main *alternative* proposals for the reported decline of stuttering: (1) The incidence has declined as an effect of public school therapy and changing attitudes in society (for example that parents are less concerned). (2) The criteria for diagnosis and reporting was influenced by the diagnosogenic theory, resulting in declining reports of stuttering. [1] These proposals will be discussed below.

Firstly, based on statistics from 147 cases of stuttering in school, Sheehan and Martyn (1970) reported that enrollment in public school therapy had no effect, positive or negative, on the probability of recovery (when controlled for the severity of the stuttering). Based on this analysis it seems unlikely that improved school therapy at this time could have had a major effect on the prevalence of stuttering. Secondly, there seems to be little support for the proposal that advice based on the diagnosogenic theory had significant effects on the prevalence of stuttering. Thirdly, it is possible that examiners influenced by the diagnosogenic theory adapted a higher threshold for the diagnosis of stuttering, thereby classifying some cases of early repetitions to be a normal behavior. However, symptoms of stuttering in school age children can hardly be considered normal according to the diagnosogenic theory. Therefore, changed criteria for diagnosis would primarily be expected to influence the diagnosis of preschool children, not school age children.

Another aspect concerns the temporal distribution of the reported decline. Firstly, the reported decline of stuttering before 1942 can not be attributed to the influence of the diagnosogenic theory. Regarding the later period it would be of interest to get more detailed accounts of how Johnson's theory was received among persons working in public schools. Did the theory have profound

---

[1] I want to thank one of the peer reviewers for this interesting proposal.

influence already in 1945? Secondly, the compilation of national data by Dean and Brown (1977) shows no indications of decline from 1964 to the end of the data series at 1973. If Johnson's theory led to reduced reporting, would this development halt in 1964? Thirdly, if Johnson's theory would have had a profound national influence on the practices of diagnosis and reporting of stuttering from public schools, it would be expected to be a factor that was known by Van Riper. However, this is not mentioned by Van Riper when discussing these results (Jackson, 1967, as reproduced in Van Riper, 1982, p. 50).

Overall, it can not be excluded that changes in attitudes and theory affected diagnosis and reporting of stuttering to some extent after the introduction of the diagnosogenic theory. However, considering the exceptionally high correlation between changes in datasets for stuttering and rheumatic fever, and the strong data from historical medical records, it is here proposed that changes in attitudes and theory had marginal effects compared with the effects of change in GAS infections.

References

Bloodstein, O., and Bernstein-Ratner, N. (2008). *A handbook on stuttering*. Clifton Park, NY: Delmar Learning.

Dean, C. R., and Brown, R. A. (1977). A more recent look at the prevalence of stuttering in the United States. *Journal of Fluency Disorders* 2, 157–166. doi:10.1016/0094-730X(77)90019-5.

Sheehan, J. G., and Martyn, M. M. (1970). Stuttering and its disappearance. *J.Speech Hear.Res.* 13, 279–289. doi:10.1044/jshr.1302.279.

Van Riper, C. (1982). *The nature of stuttering*. Englewood Cliffs, NJ: Prentice-Hall.
